# Validation of the French version of the Pittsburgh Sleep Quality Index Addendum for posttraumatic stress disorder

**DOI:** 10.3402/ejpt.v4i0.19298

**Published:** 2013-09-12

**Authors:** Malik Ait-Aoudia, Pierre P. Levy, Eric Bui, Salvatore Insana, Capucine de Fouchier, Anne Germain, Louis Jehel

**Affiliations:** 1 Centre du Psychotrauma, de l'Institut de Victimologie, Paris, France; 2 Laboratoire de Psychopathologie et Neuropsychologie (EA 2027), Université Paris 8, Vincennes - Saint-Denis, France; 3 Unité de Psychiatrie et Psychotraumatologie, Centre Hospitalier Universitaire Tenon, AP-HP, Paris, France; 4 Département de Santé Publique, Centre Hospitalier Universitaire Tenon, AP-HP, Paris, France; 5 INSERM, U707, Paris, France; 6 Université Paris 6 - Pierre et Marie Curie, UMR-S 707, Épidémiologie, Systémes d'Information, Modélisation, Paris, France; 7 Massachusetts General Hospital, Harvard Medical School, Boston, USA; 8 Laboratoire du Stress Traumatique (LST), Université de Toulouse et CHU de Toulouse, France; 9 Department of Psychiatry, University of Pittsburgh School of Medicine, USA; 10 Department of Psychiatry and Psychotraumatology, Matinique University Hospital (CHUM) Martinique, Fort-de-France Cedex, Martinique; 11 INSERM, Paris-Sud University and Paris-Descartes University, Paris, France; 12 UFR des sciences médicales, Université Antilles-Guyane, UAG, France; 13 Laboratoire d’éthique Médicale et de Médecine Légale, Paris, France

**Keywords:** Nightmares, posttraumatic sleep disturbances, trauma-related disorders, PTSD, psychometric properties

## Abstract

**Background:**

Sleep disturbances are one of the main complaints of patients with trauma-related disorders. The original Pittsburgh Sleep Quality Index Addendum for PTSD (PSQI-A) is self-report instrument developed to evaluate posttraumatic stress disorder (PTSD)-specific sleep disturbances in trauma-exposed individuals. However, to date, the PSQI-A has not yet been translated nor validated in French.

**Objective:**

The present study aims to: a) translate the PSQI-A into French, and b) examine its psychometric properties.

**Method:**

Seventy-three adult patients (mean age=40.3 [SD=15.0], 75% females) evaluated in a specialized psychotraumatology unit completed the French versions of the PSQI-A, Pittsburgh Sleep Quality Index (PSQI), Hospital Anxiety and Depression Scale (HADS), and Impact Event Scale-Revised (IES-R).

**Results:**

The French version of the PSQI-A showed satisfactory internal consistency, inter-item correlations, item correlations with the total score, convergent validity with PTSD and anxiety measures, and divergent validity with a depression measure.

**Conclusion:**

Our findings support the use of the French version of the PSQI-A for both clinical care and research. The French version of the PSQI-A is an important addition to the currently available instruments that can be used to examine trauma-related sleep disturbances among French-speaking individuals.

Sleep disturbances are common clinical problems reported by patients with psychiatric conditions, such as mood disorders, anxiety disorders, and psychotic disorders. It is estimated that over 70% of individuals with posttraumatic stress disorder (PTSD) experience sleep disturbances (Ohayon & Shapiro, 2000). Furthermore, sleep complaints are identified as a core symptom of PTSD (Spoormaker & Montgomery, 2008) and are suggested to be the hallmark of PTSD (Germain, 2013; Ross, Ball, Sullivan, & Caroff, 1989). A body of evidence suggests that trauma-related sleep disturbances reflect a central dysfunction that might contribute to the maintaining and/or worsening of PTSD symptoms (Babson & Feldner, 2010). For instance, sleep disturbances occurring after trauma exposure are associated with an increased risk for PTSD (see Germain, Buysse, & Nofzinger, 2008, for review; Mellman, Bustamante, Fins, Pigeon, & Nolan, 2002) and comorbid depression (Kessler, Sonnega, Bromet, Hughes, & Nelson, 1995). However, trauma-related sleep disturbances are still often considered as a secondary symptom of PTSD and rarely assessed in clinical settings and may therefore be underestimated (Krakow et al., 2001a; Spoormaker & Montgomery, 2008).

The most commonly experienced trauma-related sleep disturbances are nightmares and insomnia (Breslau et al., 2004). Accordingly, these sleep symptoms are included in both the re-experiencing (i.e., “recurrent distressing dreams of the event”) and hyperarousal (i.e., “difficulty falling or staying asleep”) DSM-IV-TR symptom clusters (American Psychiatric Association, 2000). Other disruptive nocturnal behaviors including night terrors, panic attacks during sleep, bad dreams not related to the trauma, and simple or complex motor behaviors during sleep are also common in PTSD (Germain, Hall, Krakow, Shear, & Buysse, 2005).

First-line psychotherapeutic interventions have been found to be efficacious in reducing daytime PTSD symptom severity. However, data suggest that they provide little to no benefit in reducing trauma-related sleep disturbances (Germain, Shear, Hall, & Buysse, 2007). For instance, it has been reported that almost 50% of patients receiving cognitive behavioral therapy for PTSD continue to experience residual sleep disturbances posttreatment (Zayfert & DeViva, 2004). Pharmacological treatments for PTSD have also been found efficacious in reducing daytime PTSD symptoms but common side effects include increased sleep disturbances (Babson & Feldner, 2010; Mayers & Baldwin, 2005).

Pharmacological (Germain et al., 2012; Raskind et al., 2007) and psychological sleep-focused interventions (Forbes et al., 2003; Krakow et al., 2001b; Moore & Krakow, 2010) have been shown to improve both nocturnal and daytime PTSD symptoms concurrently (e.g., Augedal, Hansen, Kronhaug, Harvey, & Pallesen, 2013; Escamilla, LaVoy, Moore, & Krakow, 2012). As such, prazosin (an α-1 adrenergic antagonist) and imagery rehearsal therapy (IRT) have been recommended as the treatment of choice (level A) for posttraumatic nightmares by the American Association Sleep Medicine commissioned task force (Aurora et al., 2010). While successful implementation and further examination of the efficacy of these treatments are necessary worldwide, to date, there are no instruments measuring trauma-related sleep disturbances validated in French.

Several instruments have been developed to measure sleep quality and sleep disturbances. The Pittsburgh Sleep Quality Index (PSQI) is one of the most widely used measurement instruments in clinical and research settings and has been translated into 48 different languages (Buysse et al., 2008). The PSQI was developed by Buysse and colleagues in 1989 to measure subjective sleep quality during the previous month. Although the PSQI assesses seven sleep components, it does not measure PTSD-specific sleep disturbances. For this purpose, the PSQI Addendum for PTSD (PSQI-A) was introduced by Germain and colleagues in 2005. The PSQI-A has good psychometric properties, and a cutoff score of 4, exhibits high sensitivity, specificity and a good positive predictive value for discriminating between women (Germain et al., 2005), as well as male military veterans (Insana, Hall, Buysse, & Germain, 2013), with and without PTSD. Thus, the PSQI-A provides a valid and clinically relevant measure to assess posttrauma sleep disturbances. The clinical usefulness of the PSQI-A has been recognized and the instrument has therefore been translated into other languages for broader dissemination, which includes Persian for use in Iran (Farrahi, Nakhaee, Sheibani, Garrusi, & Amirkafi, 2009).

However, to date, the PSQI-A has not yet been translated nor validated in French. Thus, this study aims to translate the PSQI-A into French and subsequently evaluate its psychometric properties. We hypothesized that the French version of the PSQI-A would demonstrate similar psychometric properties and validity compared to the original English version.

## Method

### Participants

Seventy-three adult patients who were treated at the Psychotraumatology Unit of Tenon University Hospital (Paris, France) between October 2007 and September 2008 were recruited to participate in this study. Data were collected as part of our routine clinical care. Although this research was not formally submitted to an ethics committee, it was conducted in accordance with the guidelines of the Helsinki declaration. All participants provided verbal informed consent for use of their data in this study. Participants were ≥18 years old, French speaking, had been previously exposed to traumatic events, were free from psychotic or cognitive disorders, and were hospitalized or receiving outpatient treatment. Participants had anxiety or depression, with or without PTSD symptoms and sleep disturbances.

### PSQI-A translation

First, the original English version of the PSQI-A was translated into French by L. Jehel, M. Ait-Aoudia, and C. Stancu. Step one of the English-to-French translation procedure consisted of three translations of the instrument followed by consensus agreement among the translators.

Second, the French-translated PSQI-A was back-translated into English by D. Morali, a bilingual psychiatrist who was not familiar with the original English version of the PSQI-A.

Third, both the original English PSQI-A and the back-translated French-to-English PSQI-A versions were reviewed by a panel of professionals affiliated with the Psychiatry Unit at Tenon University Hospital. The panel consisted of professionals who were involved in the first and second steps of the procedure, and others who were naive to both the English- and French-translated PSQI-A versions. At the end of this review, only small changes were made to clarify and optimize the French-translated PSQI-A. The final French version of the PSQI-A (PSQI-A FV), as presented in Appendix, was approved by several bilingual clinicians including A. Germain, a co-creator and lead author of the original (English) PSQI-A validation study.

### Measures

#### The Pittsburgh Sleep Quality Index

The French version of the PSQI was used to assess sleep quality during the past month (Blais, Gendron, Mimeault, & Morin, 1997). The 19-item self-report PSQI evaluates seven sleep components: (1) sleep quality, (2) sleep latency, (3) sleep duration, (4) habitual sleep efficiency, (5) sleep disturbance, (6) use of sleeping medication, and (7) daytime dysfunction. Each of the seven components is rated on a 0–3 scale. The total score resulting from the sum of the seven components ranges from 0 to 21, and a cut off >5 has been found to reflect clinically significant sleep disturbances (Buysse, Reynolds, Monk, Berman, & Kupfer, et al., 1989). The PSQI is one of the most frequently used instruments to examine sleep for clinical and research purposes (Buysse et al., 2008). The PSQI was translated into French and validated in 1997 by Blais and colleagues (Blais et al., 1997).

#### The Pittsburgh Sleep Quality Index Addendum for PTSD

The PSQI-A was the first validated instrument introduced to assess past-month sleep disturbances that are specifically associated with PTSD (Germain et al., 2005). The PSQI-A consists of seven items that estimate the frequency of: (1) hot flashes; (2) general nervousness; (3) memories or nightmares of a traumatic experience; (4) anxiety or panic, not related to traumatic memories; (5) bad dreams not related to traumatic memories; (6) episodes of terror or screaming during sleep without fully awakening; and (7) episodes of “acting out” dreams, such as kicking, punching, running or screaming. Each item is rated on a Likert scale from 0 to 3 (0=*not during the past month*, 1= *less than once a week*, 2=*once or twice a week*, 3=*three or more times a week*). A total score is obtained from the sum of all seven items, and has a range of 0–21. Three additional questions probing information about anxiety, anger-accompanying memories or nightmares of a traumatic experience (item 3), and timing when these occur are also included but are not added to the total score. The PSQI-A can be completed in less than 5 min.

The original PSQI-A validation study reported a Cronbach's coefficient *α* of 0.85 and a mean PSQI-A item-total correlation of *r*=0.47. All item correlations were statistically significant (*p<*0.001). The PSQI-A showed moderate to strong positive correlations with the Clinician-Administered PTSD Scale (*r*=0.53, *p<*0.007) and the PTSD Symptom Scale (*r*=0.56, *p<*0.001). A score ≥4 yielded a sensitivity of 94%, a specificity of 82%, and a positive predictive value of 93% to distinguish individuals with PTSD from those without (Germain et al., 2005).

#### Impact of Event Scale-Revised

The French version of the Impact of Event Scale-Revised (IES-R) was used to evaluate PTSD symptoms during the past 7 days (Brunet, St-Hilaire, Jehel, & King, 2003). The 22-item self-report IES-R measures symptoms of intrusion, avoidance, and hypervigilance (Weiss & Marmar, 1997). Each item is scored on a 0–4 point scale, with higher scores reflecting increased PTSD symptom severity. The IES-R is a widely used instrument reported across the international literature. The IES-R French validation study reported good internal consistency with a Cronbach's *α* of 0.93 (Brunet et al., 2003). A cutoff score of ≥36 has been shown to indicate a level of PTSD symptoms severity compatible with a PTSD diagnosis (Jehel & Dayan, 2004).

#### Hospital Anxiety and Depression Scale

Participants were administered the French version of the Hospital Anxiety and Depression Scale (HADS) to evaluate depression and anxiety symptoms during the past 7 days (Lépine, Godchau, & Brun, 1985). The HADS scale includes 14 items, each of which is scored on a 0–3 point Likert scale. Higher HADS total scores indicate increased symptom severity (Zigmond & Snaith, 1983). Specific items can be separately summed to provide two 7-item subscales for depressive symptoms (HADS-D) and anxiety symptoms (HADS-A), respectively. The French version of the HADS demonstrated satisfactory psychometric validity. Subscale scores ≥10 are commonly used to indicate both anxiety and depression for the respective subscales (Lépine, 1996; Lépine et al., 1985).

### Procedure

Participants were assessed for PTSD symptoms (re-expressing, avoidance, and hyperarousal clusters) with the IES-R, and were classified into a probable PTSD (IES-*R*≥36) based on their total score. Participants also completed the French-translated PSQI-A, PSQI, and HADS.

### Statistical analysis

Quantitative variables were tested using Mann–Whitney tests and Spearman's correlation coefficients. The internal reliability of the French-translated PSQI-A calculated with Cronbach's coefficient *α* and test–retest reliability was assessed with Spearman's correlation coefficient and the intraclass correlation coefficient. Sensitivity, specificity, and positive predictive value to identify probable PTSD were computed with 95% confidence intervals (CI). A receiver operating characteristic (ROC) analysis was implemented. SPSS 17.0 and Statview 5.0 softwares were used. The significance level was set to 0.05 (two-tailed).

## Results

The total sample was composed of 73 participants. All participants were exposed to at least one traumatic event and were being treated for PTSD symptoms. Participants had been suffering from PTSD symptoms for at least 2 months, with some for more than 10 years.

The sample included 57 participants who had IES-R scores above the cutoff value of 36 (range: 37–84) and 16 participants below the cutoff value of 36 (IES-R range: 0–35).

Patients with a score ≥36 on the IES-R were classified in the probable PTSD group, while those who scored <36 were classified in the no-PTSD group. Participants’ comorbid depression and anxiety, as well as medication use, is indicated in Table 1.

**Table 1:** Sociodemographic and clinical characteristics of 73 trauma exposed treatment-seeking adult patients with and without probable PTSD

Mean age years	Total sample *N*=73	Probable PTSD group *n*=57	No-PTSD group *n*=16

	*M*=40.3 (SD=15)	*M*=39.2 (SD*=*14.3)	*M*=44.2 (SD=17.2)
Gender			
Female	55 (75%)	45 (79%)	12 (75%)
Male	18 (25%)	12 (21%)	4 (25%)
Depression*			
Yes	45 (62%)	37 (65%)	8 (50%)
No	28 (38%)	20 (35%)	8 (50%)
Anxiety*			
Yes	62 (85%)	54 (95%)	8 (50%)
No	11 (15%)	03 (5%)	8 (50%)
Sleep medication use			
Yes	48 (66%)	41 (72%)	7 (44%)
No	25 (34%)	16 (28%)	9 (56%)

* Participants with total scores ≥10 (cutoff) on each subscales HADS-A: Anxiety state-Hospital Anxiety and Depression Scale and Depression state; HADS-D: Anxiety state-Hospital Anxiety and Depression Scale.

The probable PTSD group (*n*=57) exhibited clinically significant sleep disturbances (PSQI: range; 9–21), marked trauma-related sleep disturbances (PSQI-A: range; 3–21), and moderate to high levels of anxiety and depressive symptoms. The no-PTSD group included 16 participants who exhibited poor sleep quality and sleep disturbances (PSQI: range; 2–17), some trauma-related sleep disturbance (PSQI-A: range; 2–13), and low to moderate levels of anxiety and depressive symptoms. The no-PTSD group scored significantly lower than the probable PTSD group on the global scores for all measures (all *p*<0.05). Descriptive statistics and comparisons between the two groups are reported in Table 2.

**Table 2:** Relationship between PSQI-AFV items, clinical symptoms and probable PTSD status among 73 trauma-exposed treatment-seeking adult patients

	Probable PTSD group (*n*=57)	No-PTSD group (*n*=16)		Total sample (*N*=73)	
				
	*M* (SD)	*M* (SD)	p*	Total PSQI-A score ρ**	p**
IES-R	62.25 (11.82)	21.06 (12.62)	<0.0001	0.60	<0.0001
IES-R (without 3 items related to sleep)	–	–	–	0.58	<0.0001
HADS-A	15.51 (3.29)	10.44 (3.69)	<0.0001	0.48	<0.0001
HADS-D	11.68 (4.02)	8.50 (5.09)	0.02	0.04	0.71
Total PSQI score	15.02 (3.36)	9.44 (4.10)	<0.0001	0.55	<0.0001
Total PSQI-A score	11.77 (4.65)	6.57 (3.37)	0.0001	–	–
Hot flashes	1.38 (1.32)	0.75 (0.86)	0.09	0.57	<0.0001
General nervousness	2.47 (0.85)	1.26 (0.89)	0.0006	0.52	<0.0001
Memories/nightmares of trauma	2.14 (1.06)	1.31 (1.13)	0.009	0.60	<0.0001
Anxiety/panic not related to trauma	1.79 (1.19)	0.81 (0.99)	0.004	0.67	<0.0001
Bad dreams not related to trauma	1.44 (1.13)	1.06 (1.18)	0.22	0.57	<0.0001
Episodes of terror	1.21 (1.17)	0.44 (0.73)	0.01	0.68	<0.0001
Acting out dreams	1.33 (1.23)	0.56 (0.96)	0.02	0.63	<0.0001

Note. PSQI: Pittsburgh Sleep Quality Index; PSQI-A: Pittsburgh Sleep Quality Index-Addendum; HADS-A: Anxiety state-Hospital Anxiety and Depression Scale; Depression state- HADS-D: Anxiety state-Hospital Anxiety and Depression Scale; IES-R: Impact Event Scale-Revised, without 3 items related to sleep: item 2: waking at night; item 15: trouble falling asleep; item 20: nightmares.

* Mann–Whitney tests;

** Spearman's *ρ* test correlations.

### Psychometric properties of the French version of PSQI-A

#### Internal consistency

The PSQI-A FV demonstrated adequate internal consistency with *α*=0.72. Correlations between each PSQI-A FV items and the total PSQI-A FV score ranged from *ρ*=0.52 to *ρ*=0.67, *p<*0.0001 (Table 2).

#### Convergent and divergent validity

The PSQI-A FV demonstrated convergent validity with both PTSD and anxiety symptoms. The PSQI-A FV was associated with the IES-R total score, IES-R total score after removing the three sleep items (items: 2. waking at night, 5. trouble falling asleep, and 20. nightmares), and the HADS-A. The PSQI-A FV demonstrated divergent validity with depression symptoms. The PSQI-A FV was not associated with the HADS-D, *ρ*=0.04, *p=*0.71 (Table 2).

#### Sensitivity, specificity, and positive predictive value

**Fig. 1:**
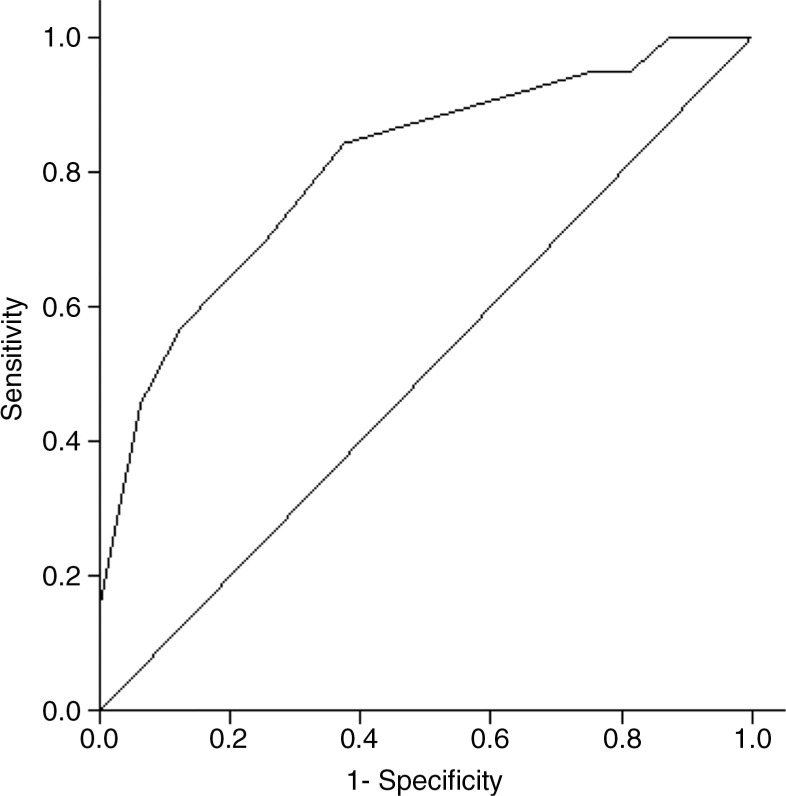
ROC curve presenting the power of the French version of the PSQI-A to discriminate between probable PTSD and no-PTSD using among 73 trauma-exposed treatment-seeking individuals.

In order to optimize the PSQI-A FV for application as a screening tool for adult patients who are most likely to develop PTSD following trauma exposure, a cutoff score was calculated to provide the highest sensitivity and strongest positive predictive value (Fig. 1). The area below the ROC curve was 0.81, showing an 81% probability that a randomly selected probable PTSD patient would have a higher PSQI-A FV score than a randomly selected no-PTSD patient. The ROC analysis also showed that a PSQI-A FV total score ≥6 would provide 87% sensitivity and 50% specificity (95% CI 79–95 and 39–61, respectively). A score ≥6 showed 86% positive and 53% negative predictive value (95% CI 78–94 and 42–64, respectively).

#### Test–retest reliability

The temporal stability of the PSQI-A FV was examined at 7-day intervals. Thirty-five patients were selected to complete the PSQI-A twice within that period, but data for only 28 patients out of the 35 were available for test–retest analyses, as seven participants did not complete the retest. Among these participants, the PSQI-A FV scores demonstrated satisfactory temporal stability, *ρ*=0.74 (*p<*0.0001) and intraclass correlation coefficient=0.76.

## Discussion

This study describes the translation of the PSQI-A to French and reports on the psychometric properties for use among French-speaking individuals. As expected, similar to the original PSQI-A, the PSQI-A FV demonstrated satisfactory internal reliability (*α*=0.85, *α*=0.72, respectively) and strong positive correlations between each item and the total score. The PSQI-A FV had good convergent validity with measures of sleep quality as measured by the PSQI. These results indicate that the PSQI-A FV items represent related aspects of a single underlying construct of sleep disturbance.

Similarly to the original English PSQI-A, the French version of the PSQI-A demonstrated good convergent validity with measures of PTSD symptom severity (*ρ*=0.60, *p<*0.001). Additionally, the probable PTSD group demonstrated a significantly higher PSQI-A FV total scores that the no-PTSD group. Thus, the PSQI-A FV total score discriminated between the probable PTSD and the no-PTSD groups, although some individuals in the no-PTSD group reported some PTSD symptoms and some PTSD sleep disturbances (see Table 2). Moreover, the correlation between the PSQI-A FV and the IES-R total score was significant even when IES-R sleep-related items were removed (*ρ*=0.58, *p<*0.001). This result suggests that the relationship between diurnal and nocturnal PTSD symptoms may be detected by the PSQI-A FV is further supported by the ROC analysis. The PSQI-A FV had an 81% ability to differentiate patients with probable PTSD from no-PTSD patients and appears to be an adequate screening instrument for probable PTSD. A PSQI-A FV score ≥6 had good sensitivity and accurately identified 87% of patients. However, the specificity of the PSQI-A FV (50%) was acceptable, albeit moderate. Additionally, 87% of patients scoring ≥6 on the PSQI-A FV total score had a probable PTSD yielding a false-positive rate of 13%. Although the cutoff score of ≥6 is higher than the cutoff score identified in the original PSQI-A validation study (≥4), the higher cutoff score identified in this study is also probably due to the different patient populations from the original study. Nevertheless, we recommend the original threshold score of 4 even when using the PSQI-A FV. This score provides a lower threshold and greater screening sensitivity to identify probable PTSD. The PSQI-A FV also demonstrated good divergent validity with a measure of depression. This suggests that PSQI-A FV is able to discriminate between dimensions of PTSD and depression.

In the original PSQI-A validation study, the total score did not change over 6 months in healthy controls group, whereas the PTSD group showed a significant reduction in the PSQI-A total score at 6 months. Our results suggest that the PSQI-A FV has good temporal stability across a short time interval. Although a 7-day interval was adequate to examine temporal stability, a longer interval would provide a more robust temporal stability assessment. Greater further analysis of sensitivity to therapeutic change would provide important information for future users of this instrument.

This preliminary study has other limitations. The sample size of this study is relatively small and our sample was composed of traumatized individuals, mostly women, who were undergoing treatment in a psychotraumatology unit. Therefore, our findings should be considered in the context of this specific sample and may not be generalized to a wider population. Further instrument examination of the psychometric properties of the PSQI-A FV in community-based samples, as well as among individuals undergoing trauma-focused treatment in various medical or mental health care settings, or individuals with other psychiatric conditions are therefore warranted. Despite these limitations, given that PTSD-related sleep problems pose therapeutic challenges (e.g., Davis & Wright, 2007; Krakow et al., 2001a, 2001c), these findings have both clinical and research implications. Within clinical settings, the PSQI-A FV is a valid tool to assess posttraumatic sleep disturbances among French-speaking individuals. Within research settings, the PSQI-A FV may be used to better understand the relationship between sleep and PTSD.

## Conclusion

The PSQI-A FV is a self-administered questionnaire that can be completed in five minutes and offers clinicians a reliable way to screen adult patients who suffer from PTSD sleep disturbances. Our study demonstrated that the PSQI-A FV has satisfactory psychometric properties among Francophone adult patients who were being treated for PTSD. Thus, the PSQI-A FV is a reliable tool, which can be used to examine posttrauma sleep disturbances and to screen for probable PTSD among trauma-exposed individuals. The validity of this instrument enables its use for both clinical and research applications. A validated version of the PSQI-A is now available for administration to French-speaking individuals.
